# Selective colonization of microplastics, wood and glass by antimicrobial-resistant and pathogenic bacteria

**DOI:** 10.1099/mic.0.001506

**Published:** 2024-10-15

**Authors:** Emily M. Stevenson, Owen Rushby-Jones, Angus Buckling, Matthew Cole, Penelope K. Lindeque, Aimee K. Murray

**Affiliations:** 1 European Centre for Environment and Human Health, Environment and Sustainability Institute, University of Exeter Medical School, Faculty of Health and Life Sciences, Penryn Campus, Cornwall, UK; 2 Faculty of Environment, Science and Economy, University of Exeter, Penryn Campus, Cornwall, TR10 9FE, UK; 3 Marine Ecology & Biodiversity, Plymouth Marine Laboratory, Prospect Place, West Hoe, Plymouth, PL1 3DH, UK

**Keywords:** antimicrobial resistance, biofilm, *Escherichia coli*, extra-intestinal pathogen, microplastic, Plastisphere, wastewater

## Abstract

The Plastisphere is a novel niche whereby microbial communities attach to plastic debris, including microplastics. These communities can be distinct from those found in the surrounding environment or those attached to natural substrates and may serve as a reservoir of both pathogenic and antimicrobial-resistant (AMR) bacteria. Owing to the frequent omission of appropriate comparator particles (e.g. natural substrates) in previous studies, there is a lack of empirical evidence supporting the unique risks posed by microplastics in terms of enrichment and spread of AMR pathogens. This study investigated selective colonization by a sewage community on environmentally sampled microplastics with three different polymers, sources and morphologies, alongside natural substrate (wood), inert substrate (glass) and free-living/planktonic community controls. Culture and molecular methods (quantitative polymerase chain reaction (qPCR)) were used to ascertain phenotypic and genotypic AMR prevalence, respectively, and multiplex colony PCR was used to identify extra-intestinal pathogenic *Escherichia coli* (ExPECs). From this, polystyrene and wood particles were found to significantly enrich AMR bacteria, whereas sewage-sourced bio-beads significantly enriched ExPECs. Polystyrene and wood were the least smooth particles, and so the importance of particle roughness on AMR prevalence was then directly investigated by comparing the colonization of virgin vs artificially weathered polyethylene particles. Surface weathering did not have a significant effect on the AMR prevalence of colonized particles. Our results suggest that the colonization of plastic and non-plastic particles by AMR and pathogenic bacteria may be enhanced by substrate-specific traits.

## Graphical abstract

Summary of key findings. Polystyrene and wood were selectively colonized by antimicrobial-resistant (AMR) bacteria, while bio-beads selected for potential *Escherichia coli* pathogens and surface weathering did not significantly influence AMR colonization. Created in BioRender. Stevenson, E. (2024) BioRender.com/r90g504

**Impact Statement**

Recent research shows that plastic debris serves as a platform for the growth of bacterial communities responsible for diseases in both humans and animals. An additional threat is posed when these diseases are unable to be treated with antimicrobials, such as antibiotics. There are reasons to suggest that the attachment of bacteria to plastics may make them more likely to become resistant to antimicrobial treatment. However, further research is required to fully understand whether plastics, specifically microplastics, pose a greater risk than natural debris in supporting these disease-causing or drug-resistant microbial communities. The findings from this study indicate that particle type affects colonization by either antimicrobial-resistant (AMR) or pathogenic (disease-causing) bacteria, with the roughness of the particle potentially having little influence. By identifying particles of greater concern for AMR risk, we can recommend improvements to waste management or sewage treatment, with the aim to reduce emissions of these materials into the environment. Policy recommendations may include proposed improvements to environmental monitoring of both microplastics and antimicrobial micropollutants.

## Data Summary

The research data supporting this publication are openly available from the University of Exeter’s institutional repository at: https://doi.org/10.24378/exe.XXXhttps://doi.org/10.24378/exe.5046.

## Introduction

Microplastics (plastic particles, 0.1 µm to 5 mm [1]) are prevalent environmental pollutants, with >120 trillion estimated to have accumulated in the global ocean [2]. Upon entering the environment, microplastics are rapidly colonized by diverse microbial communities known as the Plastisphere [3]. These communities are distinct from the surrounding environment [4,8] and, sometimes, other natural debris [9,13]. It has also been suggested that Plastisphere communities act as hotspots for human and animal pathogens [14,16] and antimicrobial-resistant (AMR) bacteria [17,20]. The transmission and persistence risk of microbes attached to microplastics may also be magnified due to the durability and widespread transport capabilities of these particles [21,23].

The colonization of microplastics by AMR pathogens is likely exacerbated by the co-occurrence of microplastics, antimicrobials, AMR pathogens and other anthropogenic pollutants in highly polluted environments, such as landfill leachates and wastewater [24,25]. Specifically, the presence of antimicrobial micropollutants and human or animal pathogens in clinical waste effluent, domestic sewage and agricultural run-off [26,30] results in the exposure of environmental bacteria to sub-inhibitory concentrations of antimicrobial compounds, which have been experimentally shown to pose an AMR evolutionary selection pressure [31,33]. Microplastic pollutants have also been widely documented to co-occur in these environments [34,36], and it has been proposed that there are microplastic-dependent characteristics that may influence the evolution and enrichment of AMR within the Plastisphere [37]. These include providing a platform for the horizontal gene transfer (HGT) of AMR genes (ARGs) [38], the concentration of AMR selective or co-selective adsorbed compounds [39] and the leaching of AMR selective or co-selective plastic additives [40].

Given the frequent co-occurrence of these pollutants in wastewater and polluted environments, it is important to understand the AMR enrichment and transmission risk posed by microplastics. Furthermore, many wastewater treatment plants (WWTPs) use plastic filters as part of their filtration process. These ‘biological aerated flooded filter’ (BAFF) plants utilize the colonization potential of plastics to generate biofilms of bacteria able to digest compounds present in sewage (e.g. ammonia) [41,42]. The risk posed by plastics adopted in sewage treatment in the enrichment and dissemination of AMR pathogens is currently unknown, but previous research has begun to reveal the role of the Plastisphere in supporting AMR pathogens in diverse environments [19,43].

Most studies, however, lack appropriate controls [44], with Plastisphere communities compared only to planktonic communities. This is problematic because of the traits naturally conferred by surface attachment phenotypes, which largely result from denser or more diverse communities and the protection provided by biofilms. The characteristics of particles that are not necessarily specific to plastics may also affect selective colonization, including hydrophobicity, crystallinity, electrostatic interactions and surface roughness [37]. For example, previous studies have found that biofilm communities are distinctively influenced by the ageing or weathering of particles [45,46]. These processes alter the surface roughness of the particle and thus increase the surface area, which has been found to influence the attachment of bacterial communities [47]. Studies should ideally compare substrate-associated and free-living communities, plastics of different polymers and both natural and inert substrate controls.

This study aims to understand how colonization of both AMR and pathogenic bacteria differs between microplastic and non-plastic substrates. Treatments included a free-living community; a natural substrate control (wood); an inert control (glass) and three types of microplastics: black, irregularly shaped and polyethylene ‘bio-beads’ used as biomedia filters in sewage treatment works; clear, smooth, polypropylene pre-production pellets (‘nurdles’) used by the plastics industry and white, expanded polystyrene balls used as packaging material. Based on the results of this experiment, a follow-up experiment was designed to investigate how artificial weathering affected colonization. In both experiments, particles were inoculated with a complex, natural sewage community, under temperature conditions that are comparable to various stages of wastewater treatment, including anaerobic digestion [48], activated sludge [49] and biological treatment reactors [50]. Whilst our laboratory conditions only loosely approximate real-world scenarios, these findings provide insights into the potential selective colonization of particles within sewerage or highly polluted environments.

## Methods

### Substrate-selective colonization

The methods described here outline the protocols used in our main study, which investigated selective colonization of AMR or pathogenic bacteria on three different types of environmental microplastics, with natural (wood), inert (glass) and free-living (liquid culture) controls.

#### Study particles

Particles were sampled, sterilized and analysed (polymer identification) as previously described by Stevenson *et al*. [51]. Briefly, microplastics comprised polyethylene ‘bio-beads’, polypropylene ‘nurdles’ and expanded polystyrene. Environmentally sampled wood particles were used as natural substrate controls. The microplastics and wood particles adopted in this study were all sampled from the same location (Truro River, UK; 50.260048–5.045549), from a 1 m^2^ quadrat using sterile forceps and rinsed with deionized water. Additionally, 4 mm glass beads (Novagen ColiRollers, LOT: D00136263) were purchased and used as inert substrate controls. To ensure the size of the study particles was controlled across particle types, only wood and microplastic particles of 4 mm in diameter (measured with a 30 cm ruler for each individual particle) were selected for further processing. With the microplastic and wood particles being environmentally sourced, there were natural differences in surface topography, which could not be controlled for, but were considered important for incorporating environmental realism. To ensure sterility of all particles, bio-beads, nurdles, wood and glass beads were autoclaved at 121 °C for 15 min. Given that expanded polystyrene is not suitable for autoclaving, these particles were gamma irradiated by Becton Dickinson (Plymouth, UK: 10.2–10.6 kGy delivered for 3600 s ×2, followed by a further 900 s). The sterility of particles was assessed by culturing particles in 10 ml Iso-Sensitest broth and shaking (180 r.p.m.) at 37 °C overnight prior to use in experiments. Sterility was confirmed for all particles where there were no visual changes in the OD of the media. Attenuated total reflectance Fourier-transform infrared spectroscopy (ATR-FTIR) was performed using a PerkinElmer 208 Spotlight 400 (Perkin Elmer, UK) to confirm the polymer type of each microplastic.

**Fig. 1.**
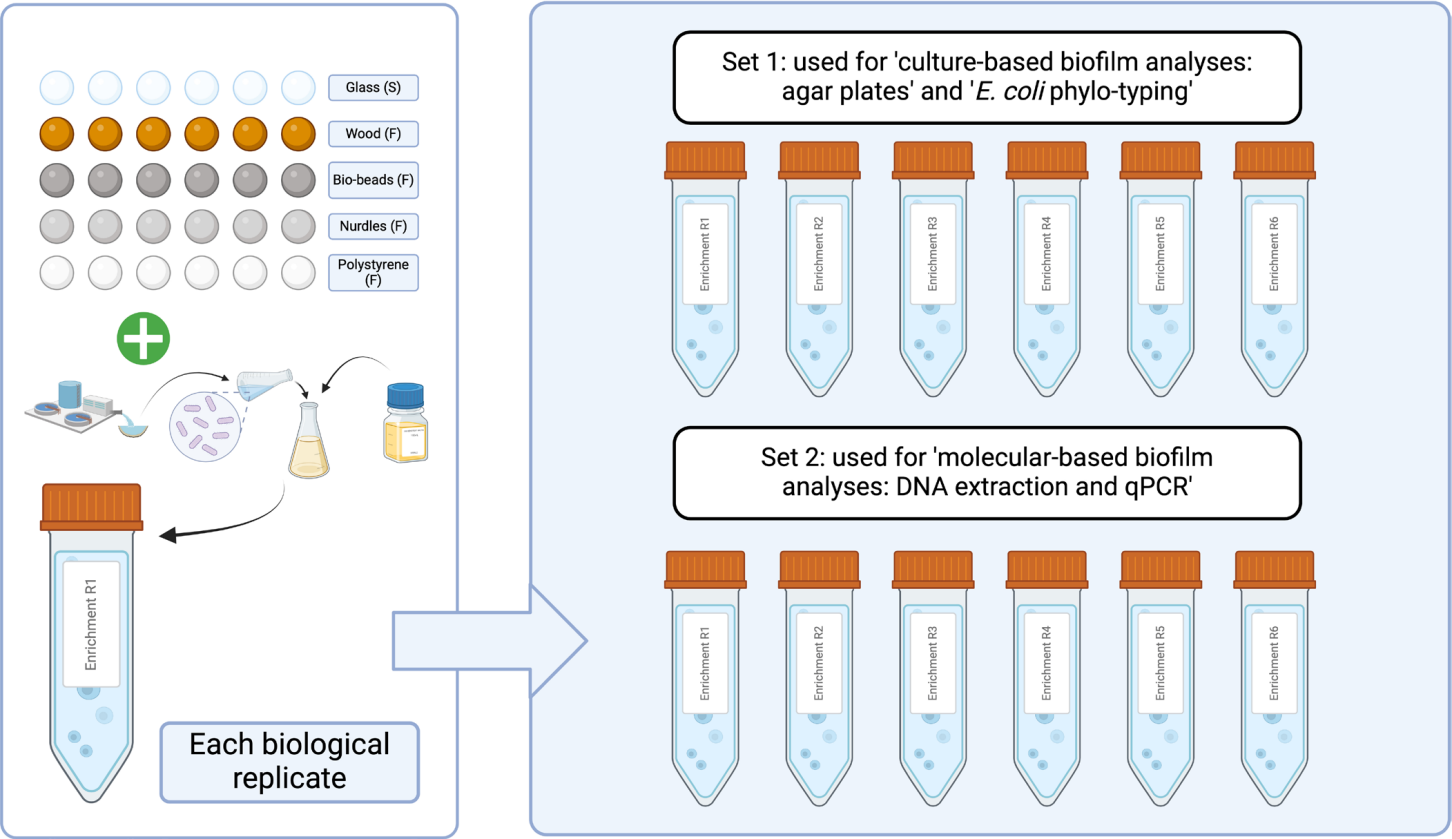
Schematic overview of particle inoculation protocol. S, particle that sunk during inoculation and F, particle that floated during inoculation. Created in BioRender. Stevenson, E. (2024) BioRender.com/q54k724

Whilst not considered as an interaction in formal analyses of the results, particles did differ in their buoyancy. Specifically, all particles except negatively buoyant glass remained floating on the surface of the microcosms (Fig. 1).

#### Particle inoculation

Sewage influent samples were collected from a wastewater treatment plant in Falmouth, UK (serving a population of ~43 000) in June 2021. The samples were transported in cool boxes, mixed 1 : 1 with 40% glycerol (ThermoFisher, LOT: P01H051) and stored at –70 °C until use. Aliquots were then thawed and spun down at 14 800 r.p.m. for 1 min, and the supernatant was discarded and the pellet was resuspended in 1 ml sterile 0.85% NaCl (Sigma-Aldrich, PCode: 1003326144) twice to remove chemical and nutrient carryover [33]. The resuspended pellet was used to inoculate at 10% (vol/vol) in 10 ml Iso-Sensitest broth (Oxoid, LOT: 3177183) in 50 ml sterile falcon tubes containing sterile particles. The Iso-Sensitest broth was chosen for use due to its low binding affinity to antibiotics [52], which is important for follow-up experiments, which will be spiked with antibiotics. Tubes were shaken at 50 r.p.m. for 20 h at 37 °C. Within each tube (i.e. ‘biological replicate’, *n*=6), there were six individual ‘particle replicates’ (i.e. technical replicates) of each particle type to account for individual variability of particle shapes and sizes (Fig. 1). This inoculation was performed twice to ensure a high enough DNA yield and a high number of culturable cells (Fig. 1), with the same nutrient medium, inoculum and conditions. Furthermore, DNA was extracted directly from the ‘Set 1’ particles using a specific DNA extraction kit for biofilms, which would not allow culturable bacteria to be obtained following the extraction process.

#### Biofilm extraction

Following the inoculation of the particles, the first set of samples was processed for direct DNA extraction from the biofilms. For this, 500 µl of liquid culture was taken and cryogenically stored with 500 µl 40% glycerol until use. The remaining, surrounding liquid culture was decanted, and particles were rinsed twice with sterile 0.85% NaCl to remove any loosely attached bacteria and left to air dry under sterile conditions inside a CAT-II safety cabinet. All six individual particle replicates for each of the six biological replicates for each particle type were then placed into 1 mL 20% glycerol and stored at –70 °C until DNA extraction.

For the particles inoculated for agar cultivation, 100 µl of liquid culture was taken and diluted with sterile 0.85% NaCl for agar cultivation. The remaining, surrounding liquid culture was decanted, and particles were rinsed twice with sterile 0.85% NaCl to remove any loosely attached bacteria and left to air dry under sterile conditions inside a CAT-II safety cabinet. Biofilms were then extracted using the extraction protocol recommended by Stevenson *et al.* [51]. Briefly, all six individual particle replicates for each of the six biological replicates for each particle type were placed into 600 µl sterile 0.85% NaCl in Eppendorf tubes. Five sterile, 4 mm glass beads were added to each tube and placed into a sonication bath (VWR Ultrasonic Cleaner, Model: USC100T) for 15 min at 45 kHz. Each tube was then vortexed (2500 r.p.m.; Scientific Industries, serial number A6. 1130) for 1 min. For agar cultivation, 100 µl of the suspended biofilm was diluted .

#### Antibiotics

Antibiotics used were ampicillin (AMP) (Sigma-Aldrich; 59349-100 MG), ciprofloxacin (CIP) (Sigma-Aldrich; 17850-25G) and trimethoprim (TRMP) (Sigma-Aldrich; 46984-250 MG). Solvents were sterile, filtered deionized water, diluted hydrochloric acid (0.1 M; Fisher Chemical; 10080210) and DMSO (100%; Sigma-Aldrich; D8418-50ML), respectively. All antibiotics were stored in single-use aliquots at −20 °C until use.

#### Culture-based biofilm analyses: agar plates

To distinguish and enumerate putative *Escherichia coli* (*E. coli*), non-*E. coli* coliforms (coliforms, e.g. *Klebsiella pneumoniae*) and non-coliforms (e.g. *Pseudomonas aeruginosa*), CM1205 Chromogenic Coliform agar (ISO) (Oxoid, LOT: 3004362) was used. The coliforms defined here are non-*E. coli*, rod-shaped, Gram-negative bacteria that are able to ferment lactose, which are largely associated with sewage [53]. Furthermore, to enable the generation of a phenotypic resistance prevalence, agar plates were supplemented with and without AMP, CIP and TRMP at clinical breakpoint concentrations for *Enterobacteriaceae* (8, 0.5 and 4 mg L^−1^, respectively; The European Committee on Antimicrobial Susceptibility Testing (EUCAST)) [54]. Biofilm suspensions were diluted using sterile 0.85% NaCl prior to plating, according to a tenfold dilution series performed to detect the optimum dilution (i.e. achieved 20–80 c.f.u. of *E. coli*). One hundred microlitres of each diluted biofilm suspension and liquid culture were plated in duplicate onto both antibiotic and non-antibiotic-supplemented agar using sterile glass beads [55]. Plates were inverted and incubated at 37 °C for 18–24 h. Colonies were then counted using a colony counter (Stuart, serial number RCC0221P160) and c.f.u. ml^−1^ was generated for each colony phenotype determined by colour: *E. coli* (blue/purple colonies), coliforms (pink colonies) and non-coliforms (colourless or cream colonies). From this, the average c.f.u. of phenotypically resistant *E. coli*, coliforms and non-coliforms was calculated.

#### *E. coli* phylo-typing

For each community type (particle associated and free-living), within each biological replicate, six *E. coli* colony replicates were selected at random from each agar treatment (no antibiotic and agar supplemented with the antibiotics AMP, CIP or TRMP). Selected colonies were spotted onto gridded chromogenic coliform agar plates with sterile toothpicks and left to grow overnight at 37 °C to ensure fresh colonies were used for PCR. A cell lysate was then produced by boiling the individual, fresh colonies in 10 mM Tris buffer (Gibco UltraPure Tris-HCl, LOT: 1790107) for 10 min at 100 °C in a PCR thermocycler (Applied Biosystems Veriti 96 well Thermal Cycler, serial number 2990228485, Model: 9902), then portioned into smaller aliquots in 96-well plates and stored at –20 °C until use.

Each lysate underwent Quadruplex PCR to enable *E. coli* phylo-typing, as previously described by Clermont *et al.* [56], using an Applied Biosystems thermocycler. The initial quadruplex reactions consist of a 25 µl volume containing 12.5 µl 2X DreamTaq Green PCR Master Mix (Thermo Fisher Scientific, LOT: 01177508), 1 µl of each appropriate primer (8 µl total) and 4.5 µl lysate. All subsequent E and C clade-specific reactions were carried out in a 25 µl volume containing 12.5 µl 2X DreamTaq Green PCR Master Mix, 1 µl of each appropriate primer (4 µl total), 4 µl nuclease-free water (Thermo Fisher Scientific, LOT: 01177508) and 4.5 µl lysate. The concentrations of primers used were 20 pmol, except for AceK.f (40 pmol), ArpA1.r (40 pmol), trpBA.f (12 pmol) and trpBA.r (12 pmol). Cycling parameters used were denaturation of 4 min at 94 °C, 30 cycles of 5 s at 94 °C and 20 s at 57 °C (group E) or 59 °C (quadruplex and group C) and a final extension step of 5 min at 72 °C [56]. Primers are listed in Table S1 and were provided by Integrated DNA Technologies (IDTDNA; London, UK).

PCR products were run on 1% agarose gel at 90 V for 1 h with GeneRuler 1 kb Plus DNA ladder (Thermo Scientific, LOT: 01076037). Using the expected size products (Table S1, available in the online version of this article), colonies (PCR products) were assigned to one of seven *E. coli* phylogenetic groups: A, B1, C, E, D, F and B2 [56]. Pathogenesis of *E. coli* is attributed to phylogenetic groups and the production of virulence factors [57]. Previously, phylo-groups B2 and D have been found to have higher virulence potential, and extra-intestinal pathogenic *E. coli* (ExPECs) are significantly more likely to be members of these lineages [56,61]. Therefore, colonies found to belong to B2 were recorded as ExPECs. Those found belonging to D or E and C or A phylo-groups underwent further PCR using the reported primer sequences and PCR conditions (Table S1). Gel electrophoresis was then performed on the remaining reactions, and colonies were assigned to the corresponding phylogenetic group. Those belonging to phylo-group D were also recorded as ExPECs.

It is important to note here that, whilst ExPECs ‘mainly belong to phylogroup B2 and to a lesser extent to group D’ [62], ExPECs may also belong to other phylogenetic groups [63,64]. Furthermore, commensal *E. coli* strains have also been identified in both phylogroups B2 and D [65]; therefore, these data should be interpreted as a putative ExPEC prevalence.

#### Molecular-based biofilm analyses: DNA extraction and qPCR

Cryogenic stores of inoculated particles and liquid culture were thawed, and DNA extraction was performed using the DNeasy PowerBiofilm kit for biofilms (Qiagen, LOT: 169048148) and the DNeasy Ultra-Clean Microbial kit for liquid culture (Qiagen, LOT: 169011985), according to the manufacturer’s instructions. These DNA samples were then diluted in 10 mM Tris buffer and used as template DNA for 16S rRNA and *intI1* qPCR.

Standard curves were generated with custom synthetic gBlocks (Table S2) provided by IDTDNA, prepared according to the manufacturer’s instructions and stored in single-use aliquots at –20 °C. gBlocks used to generate standard curves were diluted tenfold from 10^6^ to 10^2^. The efficiency of qPCR ranged from 91 to 95%, with *R*^2^ values ranging from 0.997 to 0.999. qPCR was performed using the PrimerDesign Precision Plus SYBR Green Master Mix (Z-PPLUS-R-SY-10ML), on the Applied Biosystems QuantStudio 7 Flex (serial number 278871498). Reactions consist of 10 µl Master Mix, 5 µl template, 2 µl primer (1 µl of each forward and reverse primers at 9 µM; Table S2), 0.2 µl BSA (20 mg mL^−1^; Fisher Scientific, LOT: 170419-0461) and nuclease-free water up to a final volume of 20 µl. Cycling parameters used were a 2 min initial hot start activation at 95 °C, followed by 40 cycles of data collection with 10 s at 95 °C and 60 s at 60 °C. Primers are listed in Table S2 and were provided by IDTDNA.

### Virgin vs weathered selective colonization

The methods described here outline the protocols used in our second study, which investigated selective colonization of AMR bacteria on virgin vs artificially ‘weathered’ microplastic particles.

#### Study particles

The microplastics used in the second study were 4–5 mm high-density polyethylene (HDPE) pellets (Sigma Aldrich, 427985-1 KG). Polyethylene microplastics were adopted in this study, given that we identified the Study 1 bio-beads, a particle of environmental and sewage relevance, to be polyethylene.

#### Weathering process

Microplastics were mechanically ‘weathered’ by abrasively homogenizing batches of six HDPE pellets with 1 g of fine sand collected from a beach in a PowerLyzer 24 Homogenizer (MoBio, serial number PL3 1438) set at 2000 r.p.m. for 30 s. This follows a similar protocol adopted by Sun *et al.* [66] where plastics were exposed to mechanical abrasion by sand, which promotes ‘plastic weathering and fragmentation’. Prior to use, sand was sterilized via autoclaving at 121 °C for 15 min. Particles were then vortexed in sterile, deionized water to remove any remaining sand debris and left to air dry. This resulted in an observable difference in the surface weathering of each particle before and after treatment (Fig. S1). Each batch was weighed post-treatment and compared against their pre-treatment weight to ensure that there was no change in mass (*t*_7_=−0.197, *P*=0.693, paired *t*-test) following the abrasion process. Both particle types were then autoclaved at 121 °C for 15 min whilst submerged in deionized water to avoid delamination effects.

#### Particle inoculation and biofilm extraction

Particles were inoculated similarly to those described in the previous experiment, using the same washed sewage community as the inoculum. However, as the virgin and weathered particle treatments in this study were not as distinguishable from each other as in the previous study, virgin and weathered particles were inoculated in separate tubes. Briefly, six individual sterile particles were suspended in 10 ml Iso-Sensitest broth in 50 mL sterile falcon tubes, inoculated at 10% (vol/vol). Tubes (six biological replicates) were then shaken at 50 r.p.m. for 18–24 h at 37 °C.

Following inoculation of the particles, biofilms were extracted as previously described. Briefly, 300 µl of liquid culture was aliquoted and diluted for agar cultivation. The remaining, surrounding liquid culture was decanted, and particles were rinsed twice with sterile 0.85% NaCl to remove any loosely attached bacteria and left to air dry under sterile conditions inside a CAT-II safety cabinet. Biofilms were then extracted using the extraction protocol recommended by Stevenson *et al.* [51], where all six individual particle replicates for each of the six biological replicates for each particle type were placed into 800 µl sterile 0.85% NaCl in Eppendorf tubes. Five, sterile 4 mm glass beads were added to each tube and placed into a sonication bath (VWR Ultrasonic Cleaner, Model: USC100T) for 15 min at 45 kHz. Each tube was then vortexed (2500 r.p.m.; Scientific Industries, serial number A6. 1130) for 1 min. Three hundred microlitres of the suspended biofilm were diluted for agar cultivation.

#### Culture-based biofilm analyses

Diluted biofilm suspensions and liquid culture samples were then plated following the same protocol as described above (Study 1), using the same media, technique and antibiotics.

#### Molecular-based biofilm analyses

16S rRNA and *intI1* qPCR were also carried out following a similar protocol as described above (Study 1). However, DNA was extracted from the biofilm suspensions generated from the inoculated particles and liquid culture using the DNeasy Ultra-Clean Microbial kit (Qiagen, LOT: 169011985), according to the manufacturer’s instructions. Although this method differed from the direct DNA extraction previously adopted using the DNeasy PowerBiofilm kit (Qiagen, LOT: 169048148), a previous study by Stevenson *et al.* [51] found that the 16S rRNA gene copy number in DNA samples obtained from biofilm suspensions produced by sonication and vortexing were not significantly lower than the DNA obtained from direct DNA extraction on bio-beads, nurdles, polystyrene, wood and glass pellets inoculated with the same community. 16S rRNA and *intI1* qPCR gBlocks, primers (Table S2), reagents and reaction conditions were the same as above.

### Statistics

All statistics were performed in RStudio. Colony counts were converted into c.f.u. ml^−1^. C.f.u. ml^−1^ on plates supplemented with test antibiotics were divided by c.f.u. ml^−1^ on non-selective agar plates to give a prevalence of phenotypic antibiotic resistance. Due to expected inaccuracies in agar plating, any phenotypic resistance prevalence above 1 was capped at 1.

To compare abundance data (c.f.u. ml^−1^ and 16S rRNA copy number) and AMR or ExPEC prevalence between communities, data were first tested for normality using a Shapiro–Wilks test. Where assumptions were met, ANOVA tests were used. Where data were non-normally distributed, non-parametric Kruskal–Wallis tests were used. Pairwise testing was conducted as appropriate (i.e. ANOVA followed by Tukey’s post hoc test and Kruskal–Wallis followed by Dunn’s post hoc test).

Community structure was assessed by quantifying the proportions of *E. coli*, other coliforms and non-coliforms present in each selective plate, where every colony was counted. These proportions were then modelled using a generalized linear model, where the substrate or antibiotic treatment served as a predictor and a quasi-binomial error structure accounted for overdispersion. Significant effects of specific variables were tested using pairwise emmeans tests with the ‘emmeans’ package [67]. All data were adjusted for multiple comparisons using the false discovery rate (FDR) adjustment.

## Results

### Substrate-selective colonization

#### Community abundance and diversity

**Fig. 2.**
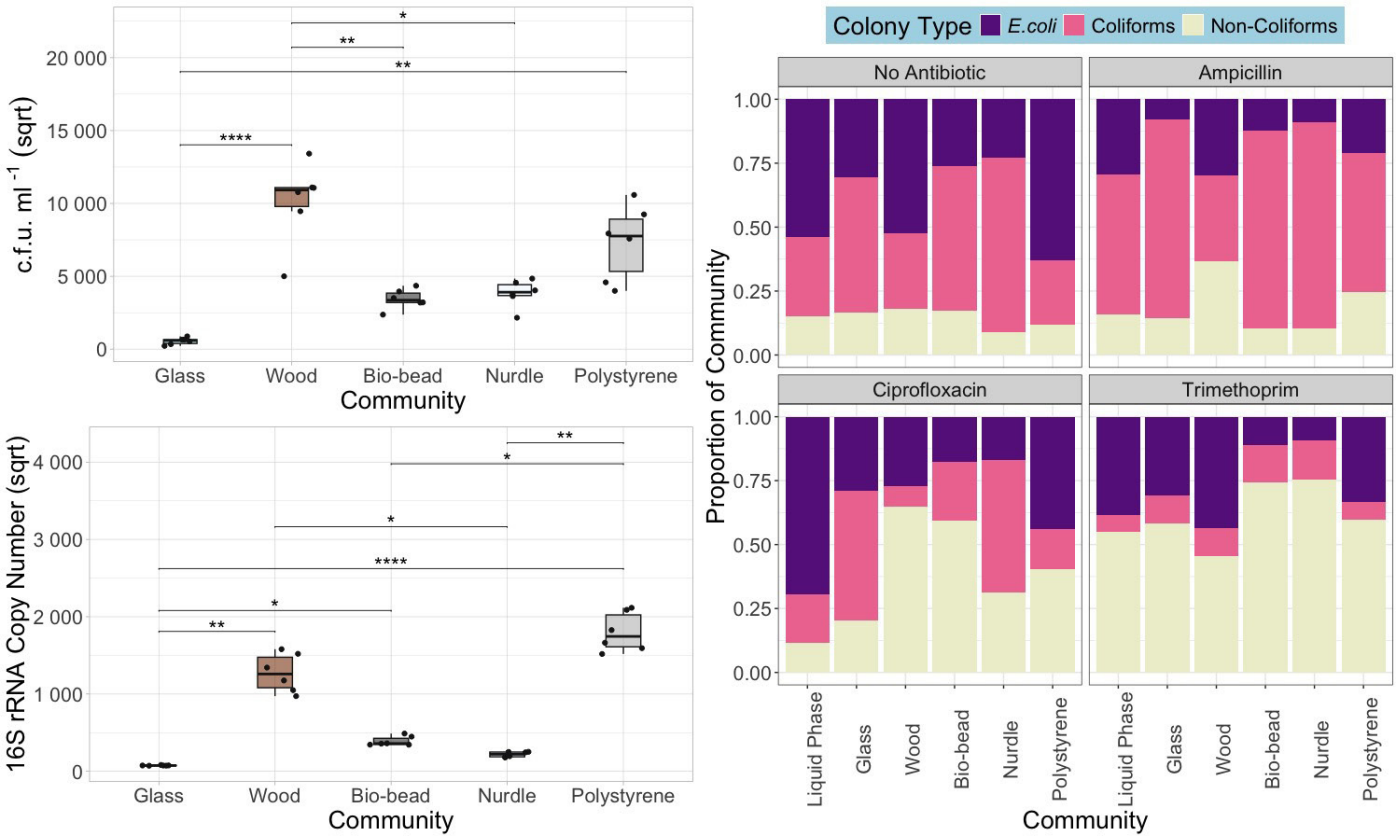
Left: total c.f.u. ml^−1^ (i.e. all colony phenotypes counted on non-antibiotic-supplemented agar plates, square root transformed) (top) and 16S rRNA gene copy number (square root transformed) (bottom) for biofilm communities (biological replicate=6). *P<0.05, **P<0.01 and ****P<0.0001 pairwise test (Kruskal–Wallis followed by Dunn’s post hoc test and FDR adjusted for multiple comparisons). Right: relative abundance of *E. coli*, coliforms and non-coliforms in cultured communities extracted from inoculated particles and liquid phase.

Using both culture-based (agar plates) and genotypic (qPCR) methods, we compared colonization by a complex sewage community across a variety of particle types. The two estimates of density, c.f.u. ml^−1^ and 16S rRNA copy number, were qualitatively consistent. Specifically, total bacterial densities differed between particle types (Fig. 2), with polystyrene and wood supporting the highest densities, based on both c.f.u. ml^−1^ (*χ*^2^ (4)=24.933, *P*<0.0001) and 16 s rRNA gene copy number (*χ*^2^ (4)=27.578, *P*<0.0001). Specifically, polystyrene c.f.u. ml^−1^ was significantly greater than glass (*Z*_4_=3.54), and wood c.f.u. ml^−1^ was significantly greater than bio-beads, glass and nurdles (*Z*_4_=2.98, 4.56 and 2.43).

16S rRNA abundance represents all bacteria present in the community (i.e. not just those able to grow on chromogenic agar). Polystyrene 16S rRNA copy number was significantly greater than glass, nurdles and bio-beads (*Z*_4_=4.66, 3.48 and 2.30), and wood 16S rRNA copy number was significantly greater than glass and nurdles (*Z*_4_=3.61 and 2.43). Given that both metrics (c.f.u. ml^−1^ and 16S rRNA copy number) revealed that polystyrene and wood were the most densely colonized particles, we can interpret these findings with greater confidence and exclude the bias of only using data on culturable vs non-culturable communities. Liquid phase data were not included in community abundance analyses due to the difficulties in comparing a volume of liquid to the surface area of solid matter.

By using chromogenic coliform agar, we were able to identify, distinguish and enumerate putative *E. coli*, non-*E. coli* coliforms (coliforms, e.g. *K. pneumoniae*) and non-coliforms (e.g. *P. aeruginosa*) by the colour of the colony. As previously detailed, putative *E. coli* were identified by purple or blue, non-*E. coli* coliforms were pink and non-coliforms formed white colonies. From this, we were able to gain an insight into the culturable community composition of each of our biofilms, with data on both resistant and susceptible strains. Community source (generalized linear model with quasi-binomial error structure, *F*_1,143_ =16.402, *P*<0.001) and presence of antibiotic (*F*_1,143_=10.308, *P*<0.001) had a significant impact on the proportion of *E. coli* (Fig. 2). Specifically, there was a significantly greater overall proportion of *E. coli* in the liquid phase (emmeans test, *Z*_5_=−6.092, *P*<0.0001; 6.511, *P*<0.0001 and −4.534, *P*<0.0001), polystyrene (emmeans test, *Z*_5_=−4.813, *P*<0.0001; −5.277, *P*<0.0001 and −3.15, *P*=0.0031) and wood (emmeans test, *Z*_5_=−4.465, *P*<0.0001; −4.940, *P*<0.0001 and −2.776, *P*=0.0092) communities than bio-beads, nurdles and glass communities.

#### Selective colonization of AMR bacteria

By using agar plates supplemented with different antibiotics, and comparing the cell counts in antibiotic-supplemented plates to plates without antibiotics, we generated a phenotypic resistance prevalence metric for the antibiotics we tested. Importantly, this method allowed the identification of both intrinsically resistant bacteria and strains with acquired AMR.

**Fig. 3.**
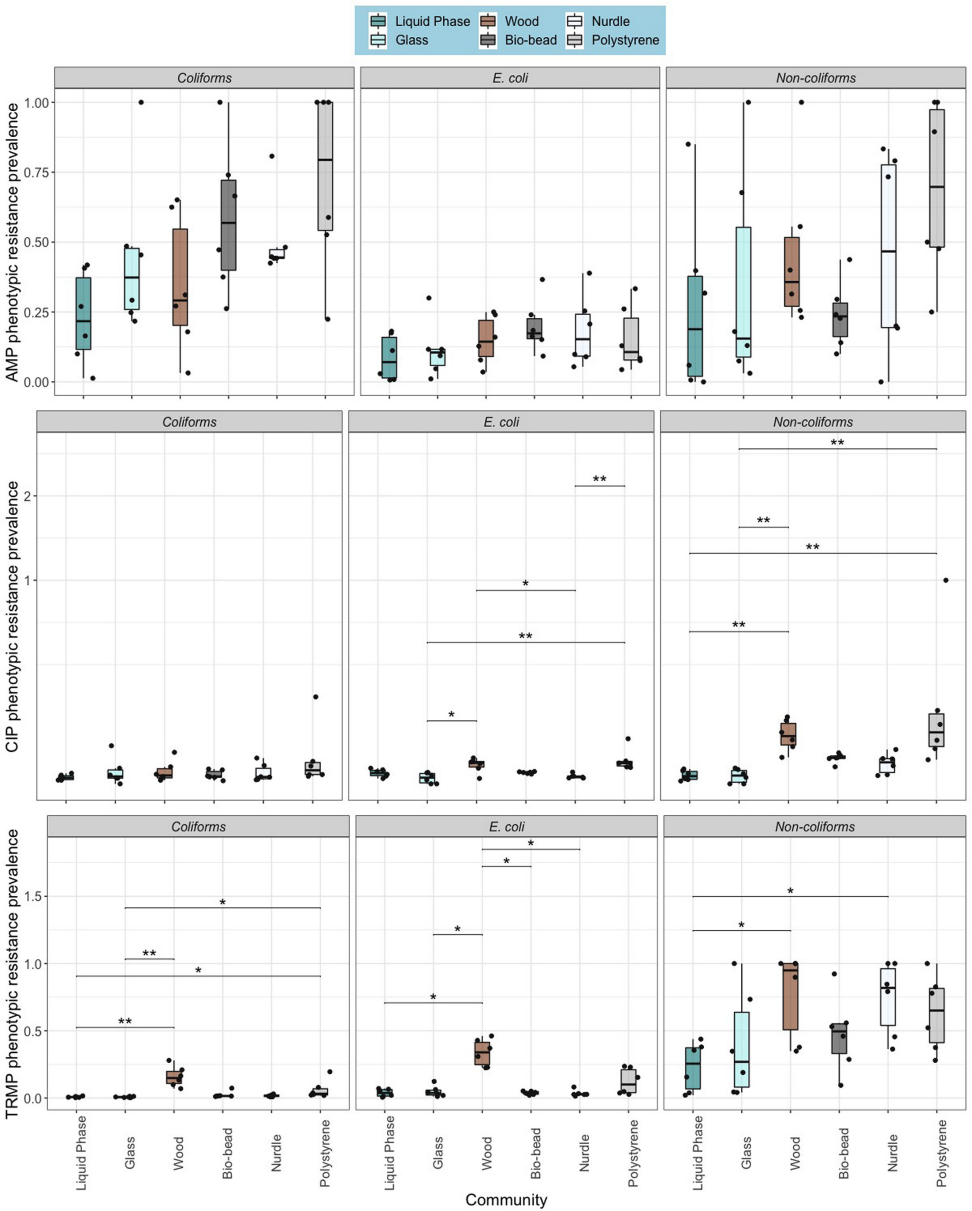
Phenotypic resistance prevalence for particle biofilm communities and liquid phase (biological replicate=6). *P<0.05 and **P<0.01 pairwise test (Kruskal–Wallis followed by Dunn’s post hoc test and FDR adjusted for multiple comparisons). Top: ampicillin, middle: ciprofloxacin and bottom: trimethoprim.

Phenotypic resistance prevalence significantly varied amongst test communities (Fig. 3), with a significant effect of community source on AMP-resistant coliforms (*χ*^2^ (5)=11.9, *P*=0.037), CIP-resistant *E. coli* (*χ*^2^ (5)=21.3, *P*=0.0007), CIP-resistant non-coliforms (*χ*^2^ (5)=27, *P*<0.0001), TRMP-resistant coliforms (*χ*^2^ (5)=26.9, *P*<0.0001), TRMP-resistant *E. coli* (*χ*^2^ (5)=17, *P*=0.004) and TRMP-resistant non-coliforms (*χ*^2^ (5)=11.6, *P*=0.0412).

For CIP, polystyrene *E. coli* communities were significantly more resistant than *E. coli* attached to glass and nurdles (*Z*_5_=3.67 and 3.43), and polystyrene non-coliforms were significantly more resistant to CIP than glass and free-living communities (*Z*_5_=3.59). Wood-associated *E. coli* were significantly more phenotypically resistant to CIP than glass and nurdle communities (*Z*_5_=2.96 and 2.71), and the CIP resistance prevalence of non-coliforms on wood was significantly greater than glass and free-living non-coliforms (*Z*_5_=3.64).

Also, polystyrene and wood communities had significantly greater TRMP resistance prevalence than glass (*Z*_5_=3.21 and 4.14) and free-living controls (*Z*_5_=3.07 and 4). TRMP-resistant non-coliforms associated with wood and nurdles were significantly enriched compared to free-living communities (*Z*_5_=2.72 and 2.61). Finally, *E. coli* associated with wood particles were found to be significantly more phenotypically resistant to TRMP than *E. coli* associated with bio-beads, glass particles, free-living communities and nurdles (*P*<0.05).

**Fig. 4.**
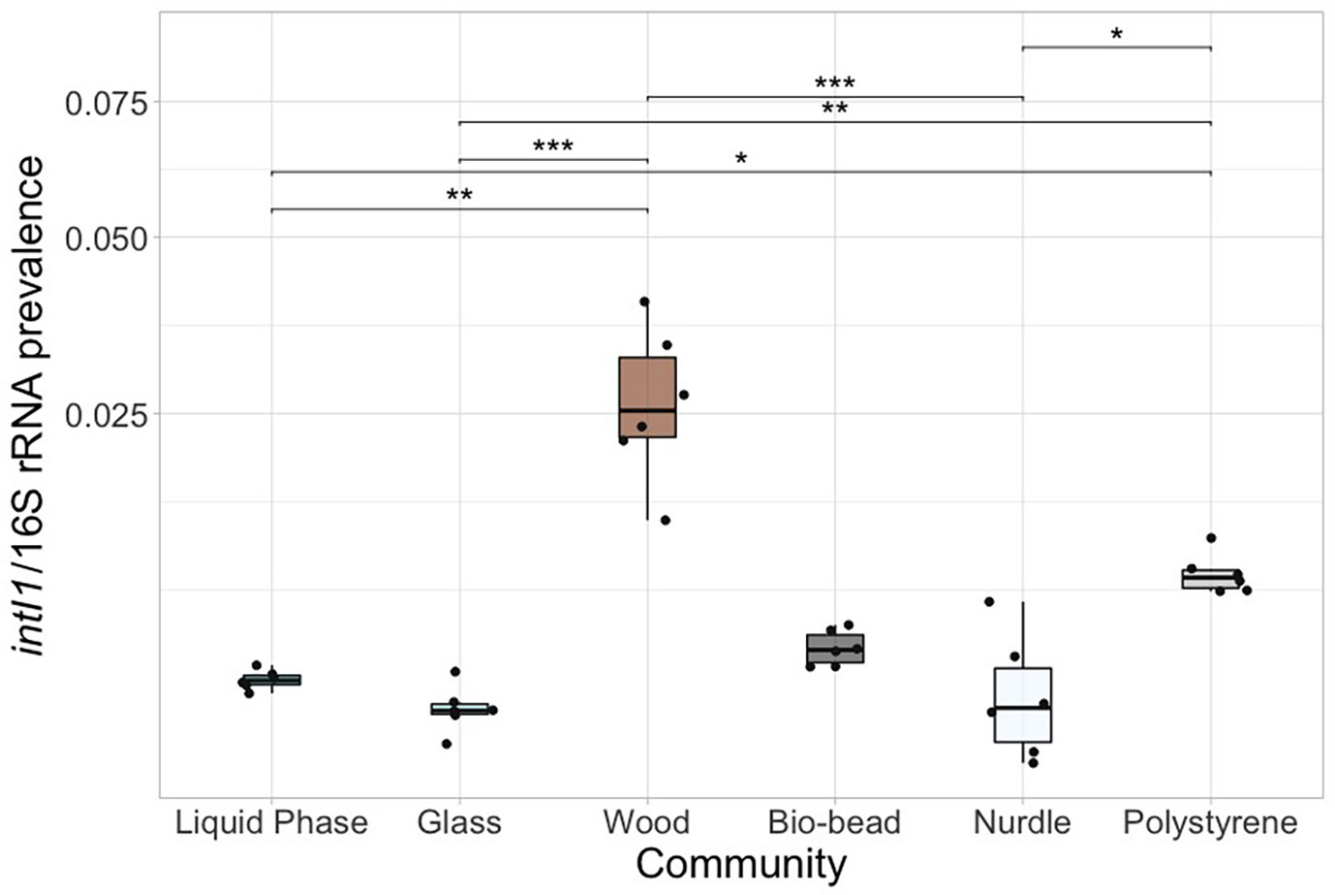
*intI1* standardized to 16S rRNA prevalence (biological replicate=6) for particle biofilm communities and liquid phase. *P<0.05, **P<0.01 and ***P<0.001 pairwise test (Kruskal–Wallis followed by Dunn’s post hoc test and FDR adjusted for multiple comparisons).

Instead of targeting a single AMR gene conferring resistance to a specified antibiotic, we used the *intI1* gene, which is often used as a proxy for AMR or anthropogenic pollution [68,71]. We found the *intI1* prevalence (standardized to 16S rRNA copy number) varied significantly between the test communities (Fig. 4; *χ*^2^ (5)=29.072, *P*<0.0001). Specifically, wood and polystyrene biofilms had significantly greater *intI1* prevalence than free-living (*Z*_5_=3.37 and 2.38), glass (*Z*_5_=4.3 and 3.32) and nurdle communities (*Z*_5_=3.89 and 2.9).

#### Selective colonization of ExPECs

**Fig. 5.**
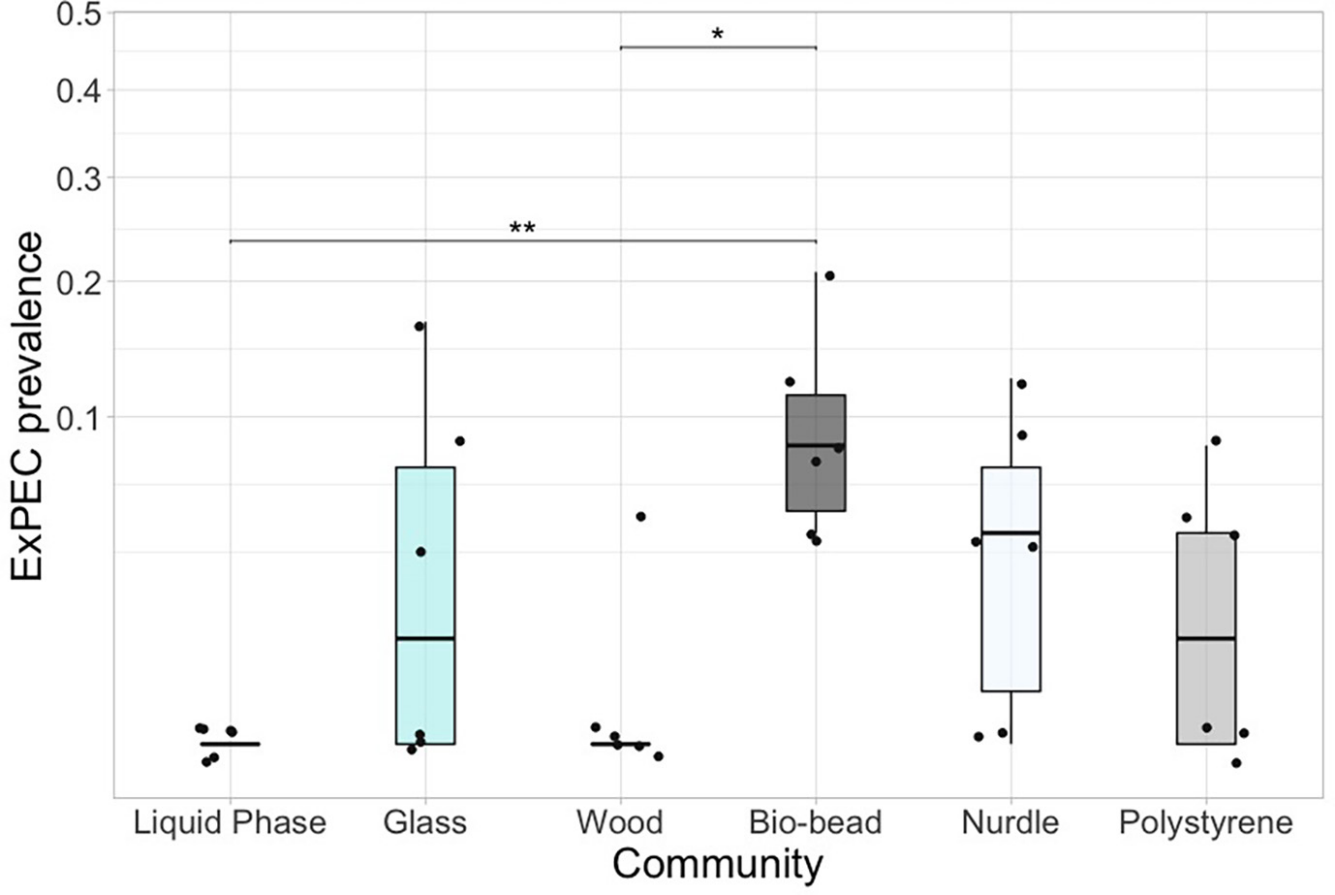
ExPEC prevalence (biological replicate=6) for biofilm communities and liquid phase. *P<0.05, **P<0.01 and *** *P*<0.001 pairwise test (Kruskal–Wallis followed by Dunn’s post hoc test and FDR adjusted for multiple comparisons)*.*

We not only investigated the abundance of *E. coli,* a widely environmentally prevalent faecal indicator species and known human pathogen responsible for multi-resistant infections in the clinic [72], but also investigated selective colonization down to the phylogenetic group level. Through this method, we are able to generate an ExPEC prevalence. Prevalence of ExPECs varied significantly across communities (Fig. 5; *χ*^2^ (5)= 15.645, *P*=0.00793), where bio-bead biofilms had a significantly greater ExPEC prevalence than free-living and wood biofilms (*Z*_5_=−3.48 and −3.09; Fig. 5).

### Virgin vs weathered selective colonization

#### Community abundance and diversity

**Fig. 6.**
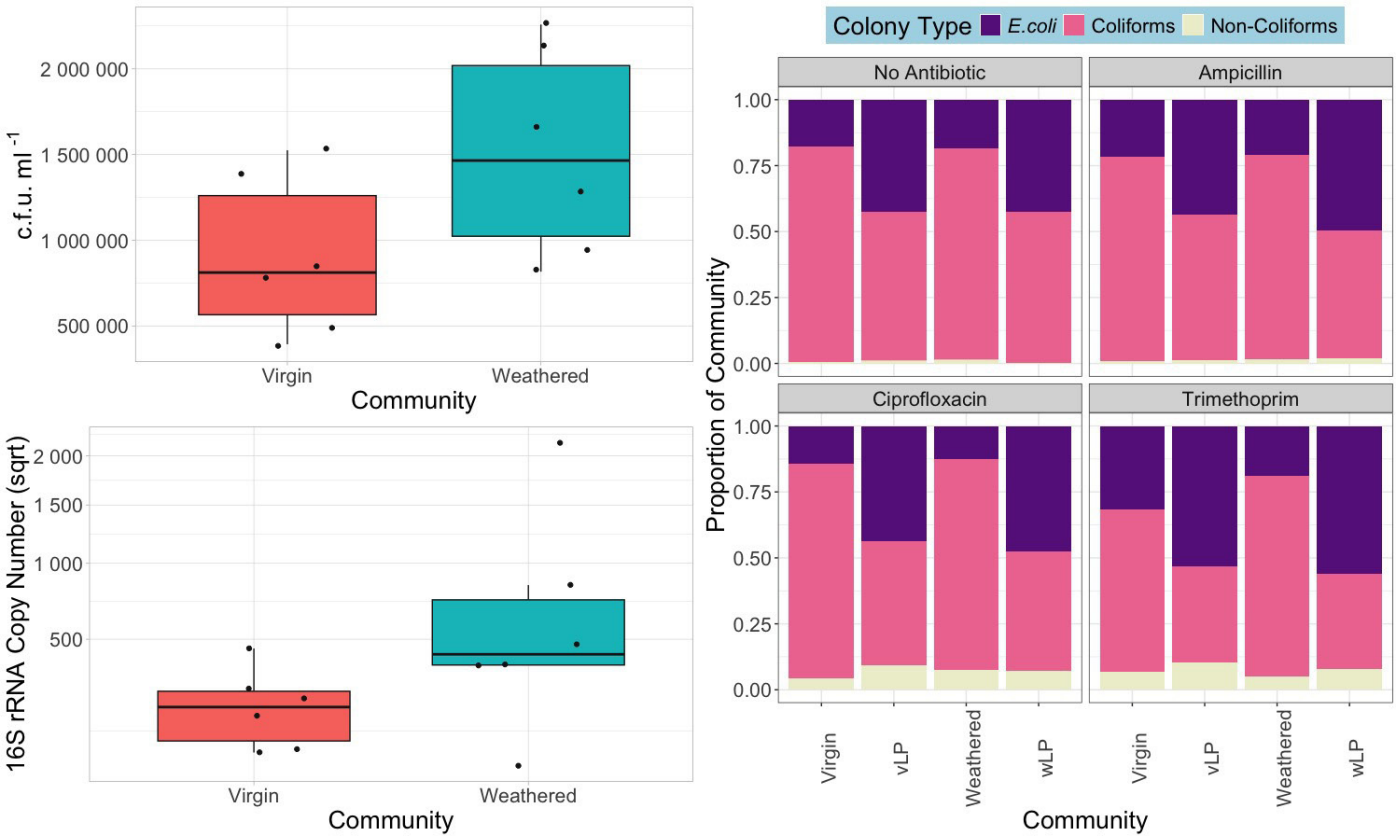
Left: total c.f.u. mL^−1^ (top) and 16S rRNA gene copy number (square root transformed) (bottom) for virgin and weathered biofilm communities (biological replicate=6). Right: relative abundance of *E. coli*, coliforms and non-coliforms in cultured communities extracted from inoculated particles and liquid phase. vLP, virgin liquid phase and wLP, weathered liquid phase.

Following our first study, we performed the same enrichment on virgin and artificially weathered HDPE microplastics. After biofilm extraction and quantification, both c.f.u. ml^−1^ and 16S rRNA copy numbers were found to be non-significantly greater on weathered particles than virgin counterparts (Fig. 6) (c.f.u. ml^−1^: ANOVA, *F*_1,2_=3.805, *P*=0.0797 and 16S rRNA: Kruskal–Wallis, *χ*^2^ (1)=2.5641, *P*=0.1093).

In terms of community diversity, there was no significant difference in the relative abundance of *E. coli* between biofilms taken from weathered and virgin microplastics (generalized linear model with quasi-binomial error structure, *F*_1,28_=0.216, *P*=0.656). However, in terms of *E. coli* abundance, each biofilm community was significantly different from their respective liquid suspension (virgin: *F*_1,46_=54.078, *P*<0.001 and weathered: *F*_1,46_=153.28, *P*<0.001) (Fig. 6).

#### Selective colonization of AMR bacteria

**Fig. 7.**
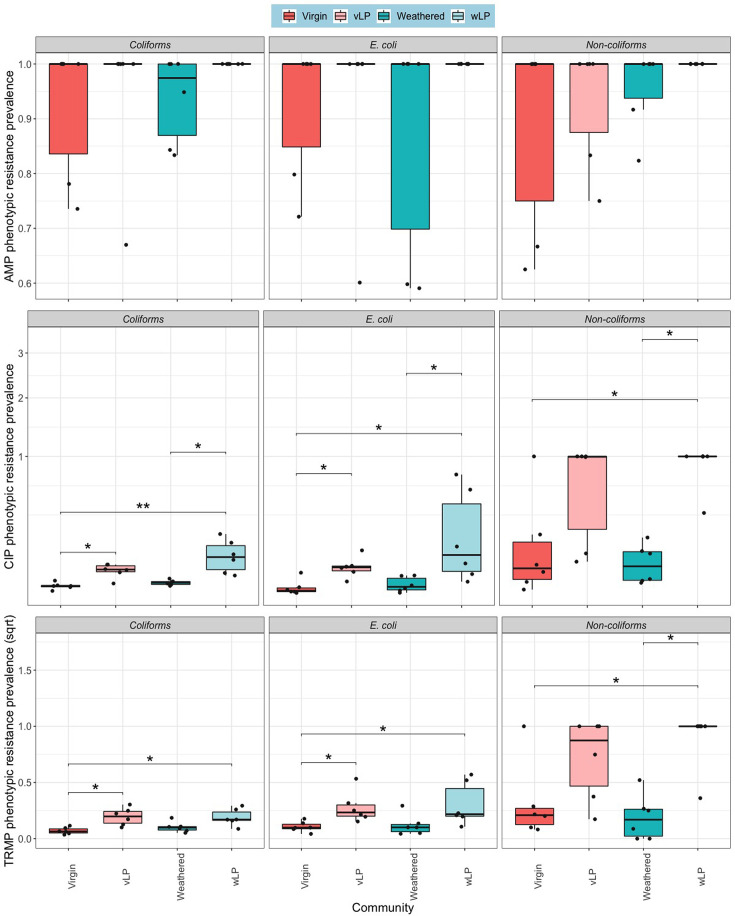
Phenotypic resistance prevalence for particle biofilm communities and liquid phase (biological replicate=6). *P<0.05 and **P<0.01 pairwise test (Kruskal–Wallis followed by Dunn’s post hoc test and FDR adjusted for multiple comparisons). Top: ampicillin, middle: ciprofloxacin and bottom: trimethoprim. vLP, virgin liquid phase and wLP, weathered liquid phase.

**Fig. 8.**
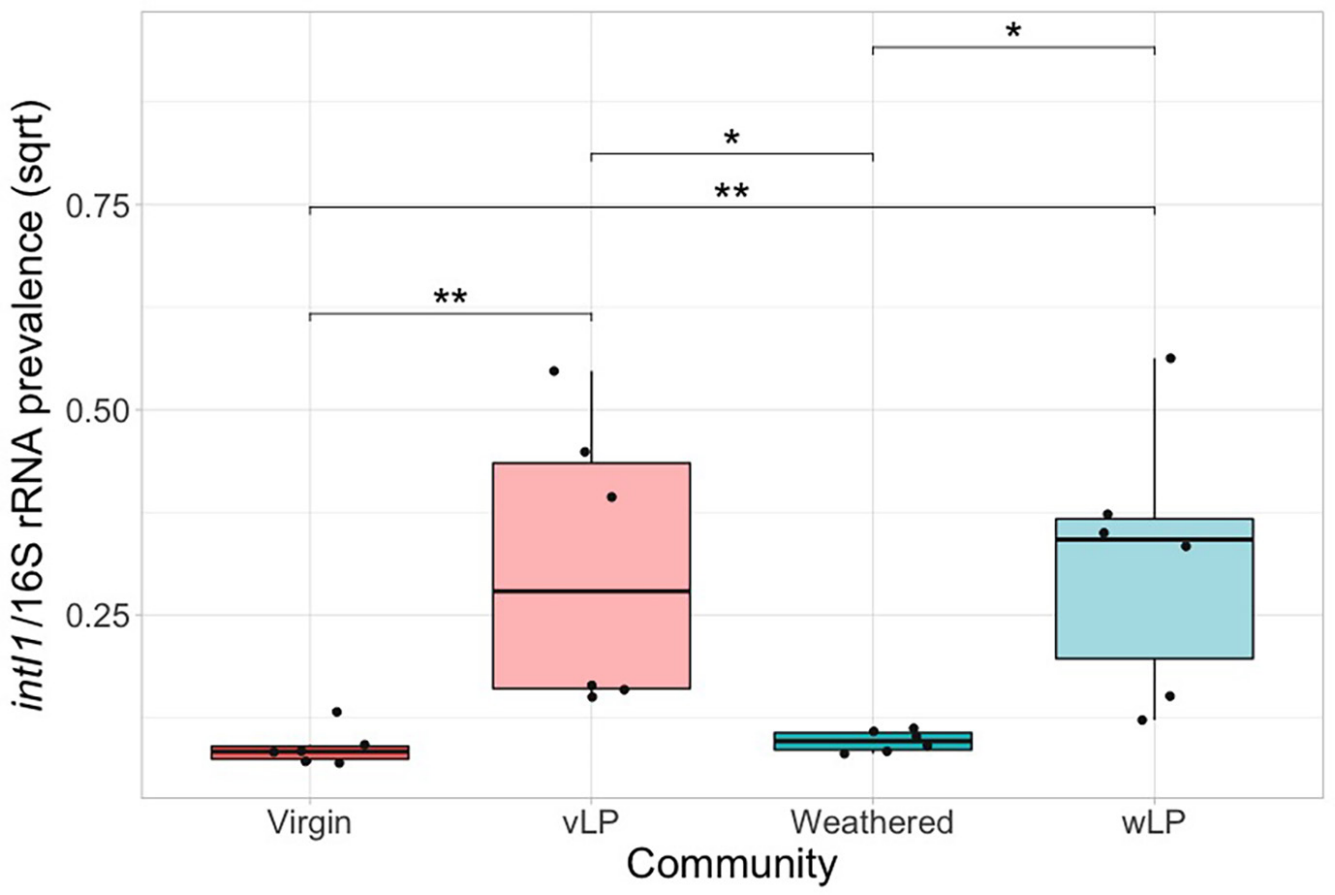
*intI1* standardized to 16S rRNA prevalence (biological replicate=6) for Plastisphere communities and liquid phase. *P<0.05 and **P<0.01 pairwise test (Kruskal–Wallis followed by Dunn’s post hoc test and FDR adjusted for multiple comparisons). vLP, virgin liquid phase and wLP: weathered liquid phase.

In most cases, phenotypic resistance prevalence was significantly greater in the free-living community compared to particles. However, the resistance prevalence in virgin and weathered Plastisphere communities did not show any significant differences (*P*>0.05, Dunn’s post hoc test; Fig. 7). Similarly, AMR (*intI1*) prevalence was significantly greater in the liquid phase in comparison to the respective biofilm communities, and there was no significant difference in the *intI1* prevalence between the virgin and weathered Plastisphere communities (*Z*_3_=0.449, *P*=0.653; Fig. 8).

## Discussion

Here, we explored how colonization by potential pathogens and bacteria carrying AMR genes was affected by substrates. Generally, the community composition of biofilms is largely driven by the external community and environment [73,74], yet here all particles (per biological replicate) were inoculated into the same environment, suggesting that the selective colonization observed is a result of substrate-specific drivers. Furthermore, given that there was no difference in the diameter of the particles adopted in this study, this also suggests that there are specific particle characteristics that differ according to particle type that affected the attachment of AMR or pathogenic bacteria. Thus, whilst the optimized laboratory conditions adopted in this study are known to select for specific strains, and in turn AMR, our results support that the colonization of plastics by AMR and pathogenic bacteria may be enhanced by particle- and polymer-specific traits.

### AMR bacteria selectively colonized wood and polystyrene

We found that polystyrene and wood biofilms enriched AMR bacteria in comparison to the other particle biofilms and free-living communities tested. These particles had the greatest community densities and greatest abundance of *E. coli* (Fig. 2), potentially due to their unique porosity allowing a greater surface area and opportunity for attachment [3,75]. As a result, an increase in complex community interactions and competition may have increased selection for AMR bacteria [76].

Competition between bacteria can increase the production of secondary metabolites, including natural antibiotics [77]. Secondary metabolite producers have been previously identified in the Plastisphere [78], with associated metabolites found to possess antimicrobial properties, which may confer a fitness advantage for AMR bacteria present. Xu *et al.* [79] found that the functional profiles involved in the metabolism of secondary metabolites were overrepresented in the Plastisphere of environmentally sampled microplastics from a lake environment in comparison to lake water samples. To further support this, the release of xenobiotics or secondary metabolites has previously been linked to an increase in HGT [80]. Specifically, mobile genetic elements (MGEs) harbouring functional genetic modules, which encode for the release of secondary metabolites, may be transferred horizontally within a community [81]. Given that we observed an increase in the prevalence of the *intI1* MGE, this may indicate an upregulation of potential transfer mechanisms within wood and polystyrene biofilms. As numerous AMR genes are also harboured on integrons, including *intI1*, this may have an indirect consequence on increased co-selection for AMR within these communities [82,84]. Competition between bacteria may also result in an increased mutation rate due to enhanced stress [76,85,89], which could also increase AMR. Conversely, selection for AMR can be reduced within a complex community, potentially due to increased costs of resistance and a community-dependent protective effect on susceptible strains [90].

Whilst we found that *E. coli* significantly dominated the polystyrene, wood and liquid phase communities, the liquid phase did not enrich for AMR. Therefore, the attachment mechanisms of *E. coli* in this community may be associated with AMR. Cellular machinery involved in surface attachment, such as adhesion factors, has been previously found to be associated with AMR, virulence and HGT [37,91]. A previous study found that sediment and rock biofilms were more likely to be associated with AMR *E. coli* than planktonic strains in the surrounding water. Maal-Bared *et al.* [92] speculated that this was likely due to the platform provided by biofilms for increased rates of HGT of ARGs, which has also been previously documented to be elevated in the Plastisphere compared to free-living and natural aggregate counterparts [38]. A further study found that the rifampicin resistance strengthened the adhesion force of *E. coli* cells and proposed that this was due to the higher levels of ATP, which facilitated the initial adhesion and resulted in biofilm growth [93]. Further investigation should now be conducted in more environmentally realistic conditions to understand the importance of these findings in natural settings.

Biodegradability of the substrate may also be important in selecting for AMR, as the provision of an energy source has previously been proposed as an important driver of environmental biofilm communities [94]. Wood is a clear candidate for this, and polystyrene has been found to enrich hydrocarbonoclastic bacteria (bacteria capable of degrading hydrocarbon bonds) [95]. Biodegradation may be further supported on polystyrene due to the amorphous nature of the particle, which is proposed to be where biodegradation is most likely to begin in biofilms [96,98]. In response to the growing threats and volume of environmental plastic pollution, alternative, non-fossil-based ‘biodegradable’ polymers have been proposed as a solution. However, recent studies have found that biodegradable plastics, including polylactic acid and polybutylene adipate-co-terephthalate, can enrich AMR bacteria over non-biodegradable microplastics [99,100]. Zhou *et al.* [99] suggest that biodegradable microplastics pose a higher risk of AMR dissemination due to higher biomass on biodegradable substrates, increased surface area due to a typically rougher surface and/or the increased bioavailability of nutrients and organic molecules from the substrate. These same traits may also be exploited by the AMR bacteria we found enriched on wood and polystyrene particles. To further support the importance of the biodegradability of the particle in attachment, glass particles were poorly colonized and did not enrich AMR bacteria. Whilst this may be due to the comparatively smooth, more homogeneous surface and lower surface area, glass is also inorganic and would not have provided an energy source to the microorganisms.

### Bio-beads significantly enriched ExPECs

Pathogens have been previously found to be enriched in the Plastisphere [3,16, 43, 75, 101,109], and whilst these strains have been identified within this microniche at genus (e.g. Oberbeckmann *et al.* [110]) and species levels (e.g. Frère *et al.* [75]), here we provide a robust identification of a focal species’ pathogenicity at the phylo-group level [56]. Pathogenesis of *E. coli* is attributed to phylogenetic groups and the production of virulence factors [57], with B2 and D lineages possessing higher virulence potential, and containing numerous ExPECs [56,61], which can cause serious infections when they enter specific areas of the body [111]. Using multiplex PCR [56], we found that polyethylene bio-beads exhibited a preferential attachment by ExPECs over free-living and natural controls (Fig. 5).

A recent study found that, in comparison to a natural substrate control, quorum sensing-related gene expression was significantly greater in microplastic biofilms [112]. Quorum sensing is essential in cell-to-cell communication [91] but is also responsible for the control of virulence factors and changes from a planktonic to a sessile cell. Furthermore, attachment phenotypes typically exhibit an increased functional diversity or a variety of metabolic responses and pathways [113,114]. This suggests that quorum sensing could have played a role in the enrichment of ExPECs observed on bio-beads; however, if so, it is unclear why the same effect was not observed on the other microplastic particles. Given that we did not see a greater proportion of *E. coli* colonizing bio-beads (Fig. 2), and yet a greater ExPEC prevalence was found, it indicates that there is something bio-bead-specific driving the selective colonization of ExPECs. Potential drivers may include contaminants associated with the bio-beads following the co-existence of these particles with mixtures of co-contaminants in the WWTPs they were originally housed in [42].

Our findings related to bio-beads are concerning in terms of environmental or human health risk assessment for a number of reasons. As noted, bio-beads are used as BAFF media in some WWTPs [42,115]. Housed in WWTP reactors, bio-beads provide a buoyant substrate, which promotes bacterial colonization, used to digest compounds including ammonia [41]. Across the UK, there are at least 55 BAFF plants. To provide some perspective on the quantity of bio-beads employed in a single plant, one UK WWTP (serving 85 000 people) holds ~43 billion bio-beads [42]. While steps are taken to limit bio-bead loss from WWTPs, significant losses do occur [115], including a spill of ~5.4 billion bio-beads in 2010 into the river system where the particles for the present study were sampled. Faecal pollution from WWTPs introduces pathogens into the environment [116], and, given that we found ExPECs were enriched on bio-beads, these particles could act as vectors and increase the spread of human or animal pathogens from WWTPs to the environment when spills occur.

Considering the global threat of multi-drug resistant, pathogenic *E. coli* [117] and source overlap of bio-beads, AMR bacteria, human or animal pathogens and other sewage-related contaminants, in combination with the findings presented here, we suggest that efforts to reduce the spill of bio-beads should be prioritized. An alternative treatment option may also be present in the use of wood in place of bio-beads, as we find that this material forms dense biofilms and enriches AMR bacteria, yet the risk posed by this organic and degradable material in the environment is significantly less than non-degradable microplastics.

### Surface weathering did not have a significant effect on AMR colonization

We hypothesized that the roughness of wood and polystyrene was responsible for the increased colonization by AMR bacteria and *E. coli.* We artificially weathered HDPE particles and inoculated them alongside virgin counterparts with the same sewage community as the first study and compared AMR prevalence. Polyethylene particles were adopted for the second study given their environmental relevance, as revealed by the ATR-FTIR identification of our environmentally sourced bio-beads as polyethylene. As the environment of focus for the present study is sewage treatment, it was important to choose a polymer that is frequently adopted for BAFF treatment. Furthermore, we found that polyethylene particles posed an enrichment risk for pathogens.

However, the findings presented here revealed no significant effect of surface weathering on the colonization of AMR bacteria to our tested microplastics. This is contrary to previous studies [98,118,121] where, in general, a greater surface area caused by an increased surface roughness results in a greater area available for colonization [122]. However, when modifications are made to surfaces on the nanoscale, this has also been found to result in less biofilm formation due to incompatibilities in size between the bacterial cell and surface pits [123,124], which may have impacted the attachment capability of bacteria in our study.

In this study, HDPE particles were artificially weathered by exposure to a short abrasion process with fine, beach sand. This process noticeably tarnished their surfaces and altered the smooth surface present on the virgin samples (Fig. 1); however, the degree of surface roughening was not quantified beyond this qualitative assessment. Importantly, previous studies reveal that a ‘surface roughness threshold’ exists, below which further decreases in surface roughness will not equate to decreases in bacterial adhesion [125].

The roughness of a surface is normally regarded as a consistent value across the entire surface of the substrate; however, microplastics can have surface blemishing, pits, indents, cracks and grooves [126,127]. A study by Hou *et al.* [128] on biofilm formation with respect to surface microtopography on a smooth substrate found that *E. coli* preferentially adhered to and formed biofilms in crevices 5–20 µm, finding that the biofilm surface coverage was more than double in crevices than on an exposed surface. We posit that such grooves increase the survivability of microbes against environmental hazards, and a heightened persistence could mean that the bacteria in these locations act as a genetic reservoir of ARGs, which the entire biofilm can draw from through HGT. Similar types of these genetic reservoir cells have been observed in complex biofilm structures before, given the name ‘persisters’, due to their ability to survive conditions that can kill the rest of the biofilm [129,130]. Therefore, whilst we found no significant difference in the AMR prevalence in this short-term study, it could be suggested that the weathered particles would house more persister cells, which could ultimately lead to the greater emergence of AMR during a longer-term study, giving time for ARGs to disseminate throughout the biofilm.

There are numerous other surface properties that can have a general impact on the rate of biofilm adhesion and, therefore, AMR prevalence. Overall, these tend to promote microbial adhesion by lowering the energy required for the physicochemical interactions that mediate the adhesion between a biofilm and its substrate [131]. These can differ significantly depending on both the adhering cell and substrate. One surface property relevant to this current study is the surface charge density. Zhu *et al.* [132] found that *E. coli* had a higher microbial attachment rate to positively charged polymer layers. Rodrigues and Saron [133] found that HDPE waste can become either negatively or positively charged through triboelectric charging, which causes static electricity by rubbing two objects together. This could be significant because our weathering method involved high-intensity rubbing of the HDPE pellets with sand. This could, theoretically, have imparted a charge on the surface of the weathered microplastic treatments and, therefore, influenced the attachment dynamics of the biofilm community. Therefore, further testing should also explore alternative weathering methods.

### Additional study limitations

Buoyancy of particles was not considered in our analyses. This may affect colonization dynamics as bacteria that favour higher oxygen levels and are capable of tolerating periods of dryness, for example, would select particles that float and remain at the air–liquid interface [134]. For example, Cheng *et al.* [135] found that the buoyancy may have influenced the colonization of their test particles: HDPE (floats) and polylactic acid (sinks). This was proposed to cause differentiation in the exposure of bacteria to oxygen, water and light intensity. In the present study, all particles floated on the surface except glass, which could explain the low colonization and AMR prevalence of these particles. However, this does not explain the selective colonization of wood and polystyrene over the other floating particles, bio-beads and nurdles. Furthermore, considering buoyancy presented an additional level of complexity that was outside the scope of this study, including the influence of colonization on substrate buoyancy. It has been found that colonization of particles, both natural and plastic, can lead to increases in particle density and reduction in buoyancy, and it would be expected that bacterial communities would change with depth, but this was not considered in the present study [136].

Importantly, we have endeavoured to increase the environmental reproducibility to the best of our abilities within the lab setting and optimal condition parameter restrictions. Notably, all microplastics were environmentally sourced, and the inoculum used to generate biofilms was an untreated sewage community, thereby increasing environmental relevance and allowing for the consideration of bacterial community dynamics, often overlooked in single-species assays. Whilst the temperature adopted in the inoculation stage of our experiment is not reproducible of surface waters in the natural environment, temperatures in various stages of wastewater treatment can reach similar levels. For example, activated sludge systems regularly reach up to 40 °C [48,49]. Furthermore, the temperature adopted in this study overlaps with the average temperature range of biological wastewater treatment, where bio-beads are typically housed. It is key to note, however, that not all WWTPs adopt a biological treatment stage, nor will they all reach these temperatures.

Finally, a high-nutrient broth, namely Iso-Sensitest broth, was chosen as the inoculation fluid to maximize growth. Whilst sewage environments, the environment in focus for this study, are known to be extremely high in nutrients [137,138], these high-nutrient conditions are not reproducible in surface waters or even in sewage effluent. Therefore, further research is required to understand how Plastisphere communities may change along the wastewater–surface water continuum. Previous studies have begun to elucidate this, for example, clinically important strains of *E. coli* have been found to survive and retain pathogenicity on plastic for at least 28 days under simulated environmental conditions, with some cases exhibiting increased virulence [139]. Moreover, microplastics and glass controls have also been found to support the persistence of potential *E. coli* pathogens for at least 25 days during simulated transitions through the freshwater–marine continuum using environmentally sampled water, demonstrating the potential for the environmental spread of pathogens on substrates [140].

## Conclusion

This study demonstrates the importance of microplastics as platforms for the attachment, enrichment and spread of AMR pathogens in the environment, and that the type of particle influences the enrichment of both AMR and pathogenic bacteria. Compared to controls, polystyrene and wood were found to enrich AMR bacteria, and bio-beads enriched ExPECs. Our data suggest that surface weathering is potentially not a significant driver in the selection of AMR bacteria, although this finding is limited by the methods used. Whilst bio-beads can be an important component of wastewater treatment, systems adopting this treatment method should prioritize monitoring the release rates of these particles and ensuring a reduction in spill risk. Furthermore, it is important that future research addresses the long-term implications of AMR enrichment on microplastics, specifically focusing on the AMR evolutionary pathways exploited by Plastisphere communities. With this, it is crucial to develop our understanding of how microplastics may influence the emergence, persistence and transmission of AMR and pathogens in the environment, as well as the subsequent risks to humans or aquatic organisms and ecosystems.

## supplementary material

10.1099/mic.0.001506 Uncited Supplementary Material 1.

## Abbreviations

AMP: ampicillin
AMR: antimicrobial resistant
ARG: AMR gene
ATR-FTIR: attenuated total reflectance Fourier-transform infrared spectroscopy
BAFF: biological aerated flooded filter
CIP: ciprofloxacin
EUCAST: The European Committee on Antimicrobial Susceptibility Testing
ExPECs: extra-intestinal pathogenic *Escherichia coli*
FDR: false discovery rate
HDPE: high-density polyethylene
HGT: horizontal gene transfer
IDTDNA: Integrated DNA Technologies
MGEs: mobile genetic elements
qPCR: quantitative polymerase chain reaction
TRMP: trimethoprim
WWTPs: wastewater treatment plants
