# Supplementary material for: Selective colonization of microplastics, wood and glass by antimicrobial-resistant and pathogenic bacteria
Source: Microbiology (Reading). 2024 Oct 15;170(10):001506. doi: 10.1099/mic.0.001506 (PMC11477370; doi:10.1099/mic.0.001506)
Supplement: Uncited Supplementary Material 1. [file mic-170-01506-s001.pdf]

Selective Colonisation of Microplastics, Wood and Glass by Antimicrobial Resistant and Pathogenic Bacteria

Author names

*Emily M Stevenson[a,b,c,], Owen Rushby-Jones[b], Angus Buckling[b], Matthew Cole[c], Penelope K Lindeque[b,c] and Aimee K Murray[a]**

Affiliation(s)

[a] European Centre for Environment and Human Health, Environment and Sustainability Institute, University of Exeter Medical School, Faculty of Health and Life Sciences, Penryn Campus, Cornwall, UK
[b] Faculty of Environment, Science and Economy, University of Exeter, Penryn Campus, Cornwall, TR10 9FE, UK
[c] Marine Ecology & Biodiversity, Plymouth Marine Laboratory, Prospect Place, West Hoe, Plymouth, PL1 3DH, UK

Corresponding author and email address

**Correspondance: es718@exeter.ac.uk (E.M. Stevenson) and a.k.murray@exeter.ac.uk (A. K. Murray)*

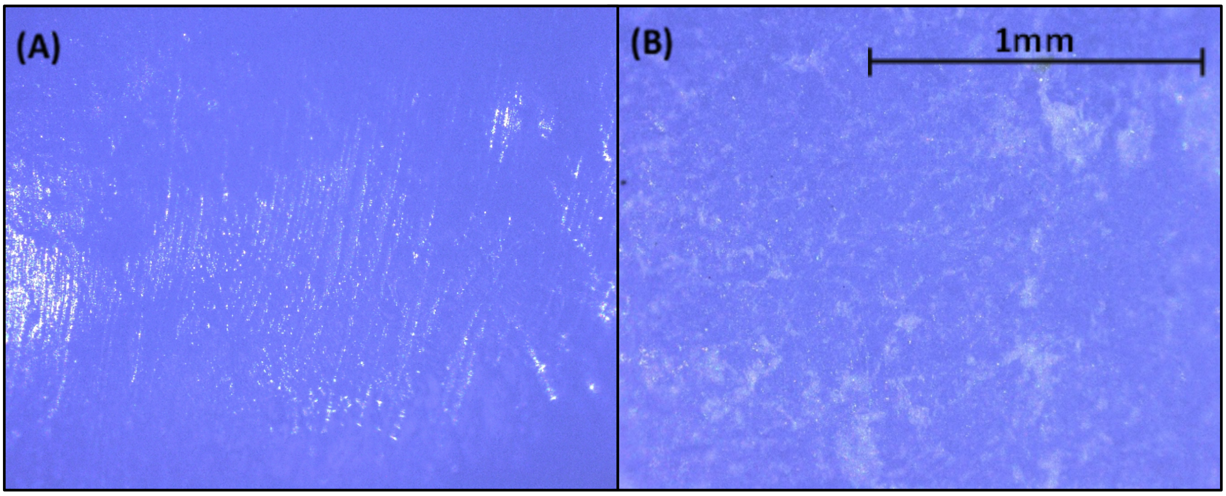

Supplementary Figure 1. Microscope images of microplastic surfaces before artificial weathering process (A) and after (B).

Supplementary Table 1. Primer sequences and sizes of PCR products used for *E. coli* phylo-typing (adapted from Clermont et al. [1]).

PCR reaction	Primer ID	Target	Primer Sequence (5' to 3')	Amplicon size (bp)	Ref
**Quadruplex**	chuA.1b	*chuA*	ATGGTACCGGACGAA CCAAC	288	Clermont *et al.* [1]
	chuA.2		TGCCGCCAGTACCAA AGACA		Clermont et al. [2]
	yjaA.1b	*yjaA*	CAAACGTGAAGTGTC AGGAG	211	Clermont *et al.* [1]
	yjaA.2b		AATGCGTTCCTCAAC CTGTG		
	TspE4C2.1b	*TspE4.C 2*	CACTATTCGTAAGGT CATCC	152	Clermont *et al.* [1]
	TspE4C2.2b		AGTTTATCGCTGCGG GTCGC		
	AceK.f	*arpA*	AACGCTATTCGCCAG CTTGC	400	Clermont *et al.* [1]
	ArpA1.r		TCTCCCCATACCGTAC GCTA		Clermont et al. [3]
**Group E**	ArpAgpE.f	*arpA*	GATTCCATCTTGTCAA AATATGCC	301	Lescat et al. [4]

	ArpAgp.r		GAAAAGAAAAAGAA TTCCCAAGAG		
**Group C**	trpAgpC.1	*trpA*	AGTTTTATGCCCAGT GCGAG	219	Lescat *et al.* [4]
	trpAgpC.2		TCTGCGCCGGTCACG CCC		
**Internal control**	trpBA.f	*trpA*	CGGCGATAAAGACAT CTTCAC	489	Clermont et al. [5]
	trpBA.r		GCAACGCGGCCTGGC GGAAG		

Supplementary Table 2. Primers and gBlocks used in this study for targeting qPCR genes.

**Gene target**	**Forward primer (5' to 3')**	**Reverse primer (5' to 3')**	**Amplicon size (bp)**	**gBlock sequence and length (bp)**	**Ref**
16S rRNA	CGGTGAATACGTT CYCGG	GGWTACCTTGTT ACGACT	142	ACGGTGAATACGTTCC CGGGCCTTGTACACAC CGCCCGTCACACCATG GGAGTGGGTTGCAAAA GAAGTAGGTAGCTTAA CCTTCGGGAGGGCGCT TACCACTTTGTGATTCA TGACTGGGGTGAAGTC GTAACAAGGTAACCG	gBlock: [6,7] Primers: [8]
intI1	GCCTTGATGTTAC CCGAGAG	GATCGGTCGAAT GCGTGT	196	CATGGCCTTGATGTTAC CCGAGAGCTTGGCACC CAGCCTGCGCGAGCAG CTGTCGCGTGCACGGG CATGGTGGCTGAAGGA CCAGGCCGAGGGCCGC AGCGGCGTTGCGCTTC CCGACGCCCTTGAGCG GAAGTATCCGCGCGCC GGGCATTCCTGGCCGT GGTTCTGGGTTTTTGC GCAGCACACGCATTCG ACCGATCCATA	[9]

References

1. Clermont, O. *et al.* (2013) The C lermont E scherichia coli phylo-typing method revisited: improvement of specificity and detection of new phylo-groups. *Environmental microbiology reports* 5, 58-65
2. Clermont, O. *et al.* (2000) Rapid and simple determination of the Escherichia coli phylogenetic group. *Applied and environmental microbiology* 66, 4555-4558
3. Clermont, O. *et al.* (2004) Characterization of an anonymous molecular marker strongly linked to Escherichia coli strains causing neonatal meningitis. *Journal of clinical microbiology* 42, 1770-1772
4. Lescat, M. *et al.* (2013) Commensal E scherichia coli strains in G uiana reveal a high genetic diversity with host-dependant population structure. *Environmental microbiology reports* 5, 49-57
5. Clermont, O. *et al.* (2008) Evidence for a human-specific Escherichia coli clone. *Environmental Microbiology* 10, 1000-1006
6. Murray, A.K. *et al.* (2018) Novel insights into selection for antibiotic resistance in complex microbial communities. *MBio* 9, 10.1128/mbio. 00969-00918
7. Murray, A.K. *et al.* (2020) The 'selection end points in communities of bacteria'(SELECT) method: a novel experimental assay to facilitate risk assessment of selection for antimicrobial resistance in the environment. *Environmental Health Perspectives* 128, 107007
8. Suzuki, M.T. *et al.* (2000) Quantitative analysis of small-subunit rRNA genes in mixed microbial populations via 5'-nuclease assays. *Applied and environmental microbiology* 66, 4605-4614
9. Barraud, O. *et al.* (2010) Quantitative multiplex real-time PCR for detecting class 1, 2 and 3 integrons. *Journal of antimicrobial chemotherapy* 65, 1642-1645
